# OP66 Assessment Of Patient And Public Involvement Reporting In Health Technology Assessment Documents In Brazil (2023 To 2024)

**DOI:** 10.1017/S0266462325101232

**Published:** 2025-12-29

**Authors:** Aléxia Vieira, Aline Rocha, Amanda Assis, Ana Pinto, Álvaro Atallah

## Abstract

**Introduction:**

Patient involvement in health technology assessment (HTA) is increasingly valued globally but remains challenging, particularly in Brazil. Reporting tools like the Guidance for Reporting Involvement of Patients and the Public Short Form (GRIPP2-SF) help assess and enhance public participation efforts. This study evaluated the reporting of HTA documents published by the National Committee for Health Technology Incorporation (CONITEC).

**Methods:**

We conducted a retrospective analysis of technical reports published by CONITEC between 1 January 2023 and 29 November 2024. We collected characterization data from reports that included the perspective of patients (PP) or public consultation (CP) item, in addition to the challenges for social participation. We used the GRIPP2-SF reporting guide, which has five items and aims to improve the quality of reporting on patient and public involvement in health and social care research. We performed a descriptive analysis of the data, presenting them as percentages and averages.

**Results:**

A total of 131 reports were evaluated, with 12,449 registered participants. Four documents did not include any social participation. Of the reports, 83 (63.3%) addressed the PP item and 48 (36.6%) addressed the PC item. The average duration of the public call was 13 days for PP and 18 days for PC. The experience report for the PP item was collected by consensus or random selection among patients. Challenges to social participation included lack of registration in the public call and failure to meet the inclusion criteria. Only the GRIPP2-SF “objectives, methods and results” items were described.

**Conclusions:**

Application of GRIPP-2F in the critical evaluation of patient involvement highlighted the importance of continuing to investigate dynamic and simple approaches to patient integration into HTA reports. Including patients after the report is completed should not be the end point but a trigger for incorporating them throughout the process in future projects.

